# Magnified Endoscopy Combined with Narrow Band Imaging of Minimal Superficial Esophageal Neoplasia—Indicators to Differentiate Intraepithelial Neoplasias

**DOI:** 10.1007/s12029-012-9395-0

**Published:** 2012-05-18

**Authors:** Yosuke Mochizuki, Yasuharu Saito, Ayako Kobori, Hiromitsu Ban, Makoto Shioya, Takashi Nishimura, Osamu Inatomi, Shigeki Bamba, Tomoyuki Tsujikawa, Mitsuaki Ishida, Akira Andoh, Yoshihide Fujiyama

**Affiliations:** 1 Division of Digestive Endoscopy, Shiga University of Medical Science, Seta-Tukinowa, Ōtsu, 520-2192 Japan; 2 Department of Medicine, Shiga University of Medical Science, Ōtsu, Japan; 3 Division of Comprehensive Internal Medicine, Shiga University of Medical Science, Ōtsu, Japan; 4 Department of Clinical Laboratory Medicine, Shiga University of Medical Science, Ōtsu, Japan; 5 Division of Mucosal Immunology, Graduate School of Medicine, Shiga University of Medical Science, Ōtsu, Japan

**Keywords:** Endoscopy, Esophageal neoplasms, Carcinoma, Squamous cell carcinoma, Narrow band imaging, Magnifying endoscopy

## Abstract

**Purpose:**

Clinical application of narrow band imaging facilitates diagnosis of esophageal neoplasia. However, no previous investigation has been conducted on magnifying endoscopy combined with narrow band imaging in detection of minimal superficial esophageal neoplasia, which is defined as neoplasia <10 mm in diameter. The aim of this retrospective study was to evaluate the usefulness of this combined technique in the differential diagnosis of minimal superficial esophageal neoplasia.

**Methods:**

Between January 2005 and November 2011, 53 minimal superficial esophageal neoplasias in 40 patients were diagnosed by screening upper gastrointestinal endoscopy with narrow band imaging at our hospital. We investigated findings including brownish dots, brownish epithelium, and demarcation line of minimal superficial esophageal neoplasia diagnosed histopathologically as low-grade intraepithelial neoplasia, high-grade intraepithelial neoplasia, and squamous cell carcinoma.

**Results:**

Significantly more brownish dots (*P* < 0.05) and brownish epithelium (*P* < 0.005) were observed in intraepithelial papillary capillary loops in high-grade neoplasia compared with low-grade neoplasia. When minimal superficial esophageal neoplasia was diagnosed as high-grade intraepithelial neoplasia or squamous cell carcinoma, sensitivity, specificity, positive predictive value, and negative predictive value were 88.9, 42.9, 44.4, and 88.2 %, respectively, for brownish dots; 94.4, 51.4, 50.0, and 94.7 %, respectively, for brownish epithelium; and 66.7, 62.9, 48.0, and 78.6 %, respectively, for demarcation line.

**Conclusions:**

The combined technique was useful in the differential diagnosis of minimal superficial esophageal neoplasia.

## Introduction

Esophageal cancer is the sixth largest cause of cancer-related death and has poor prognosis [1]. Treatment of esophageal cancer depends on the depth of cancer invasion, which reflects the risk of metastasis. Superficial esophageal cancer is defined as that with mucosal or submucosal invasion. Because of the low risk of lymph node metastasis in this type of cancer, endoscopic mucosal resection (EMR) has been established as a standard therapeutic approach for superficial esophageal squamous cell carcinoma (ESCC) within the lamina propria mucosa (LPM) [2–6]. Recent advances in endoscopic techniques for treating gastrointestinal tumor include endoscopic submucosal dissection, in which extensive lesions can be excised en bloc for ESCC. This technique has also become prevalent for treating stomach and colon cancer [7–10].

The risk of lymph node metastasis in esophageal cancer varies by the degree of depth. For example, Kodama and Kakegawa revealed an increasing rate of lymph node metastasis in superficial ESCC according to the following infiltration level: 0 % in the epithelium, 3.3 % in the LPM, 12.2 % in the muscularis mucosa, 26.5 % in the upper submucosal third, 35.8 % in the middle submucosal third, and as high as 45.9 % in the lower submucosal third [4]. From the viewpoint of the risk of lymph node metastasis, early detection of ESCC is vital.

Previous reports have demonstrated the usefulness of endoscopic screening combined with iodine staining in the identification of esophageal cancer [11, 12]. In recent years, narrow band imaging (NBI) combined with magnifying endoscopy (ME), a technique developed by Olympus Optical Co., Ltd., Tokyo, Japan, has created a new diagnostics method that involves observing capillaries and fine tissue structures. Clinical application of this combined technique (ME-NBI) facilitates diagnosis of esophageal neoplasia and diagnostic accuracy of invasion depth [13–18]. Morphological changes in the intraepithelial papillary capillary loop (IPCL) are important in the diagnosis of ESCC. Visualizing these changes by ME-NBI has been reported to contribute to the diagnosis of depth of invasion [19]. NBI clearly identifies esophageal epithelial tumor as a brownish lesion, and it is considered superior to iodine staining. It improves detection of neoplastic lesions of the esophagus in screening endoscopy even without ME. However, the combination of these two techniques holds promise for future clinical applications. NBI is expected to be widely used in future diagnoses of esophageal cancer. However, just as the treatment of small lugol voided lesions is completely unknown, there is no consensus for treatment of small lesions identified as brownish areas on NBI. The aim of this study is to evaluate the ability of ME-NBI to distinguish lesions <10 mm in diameter, which we defined as minimal superficial esophageal neoplasia (MSEN).

## Patients and Methods

### Patients

Our retrospective investigation examined 53 MSEN lesions <10 mm in diameter in terms of tumor size, location, morphology, use of ME-NBI, and histopathological findings. These lesions were diagnosed by screening upper gastrointestinal endoscopy with NBI in 40 patients and were resected by EMR at our hospital from January 2005 to November 2011.

### EMR Methods

EMR was performed under sedation by periodic intravenous administration of midazolam (0.05–0.1 mg/kg) and propofol (2 mg/kg/h) while monitoring blood pressure, heart rate, and blood oxygen saturation. The following endoscopes were used: GIF-Q240, GIF-Q240Z, GIF-Q260Z, GIF-H260, and GIF-Q260J (Olympus Optical Co., Ltd., Tokyo, Japan). After the lesion was confirmed by NBI, the oral and anal sides of the lesion were marked using a needle knife and high-frequency devices (VIO300D; ERBE, Elektromedizin GmbH, Germany). After local injection of saline into the submucosal layer, the lesion was aspirated through the end cap attached to the endoscope. Subsequently, the aspirated lesion was removed using the snare EMR method in the Endocut mode of the high-frequency device. To control bleeding from exposed vessels or the ulcer site after resection, hemostatic treatment using a hemostatic forceps was administered (FD-410LR; Olympus) by the soft coagulation mode.

### Endoscopic Findings

**Fig. 1:**
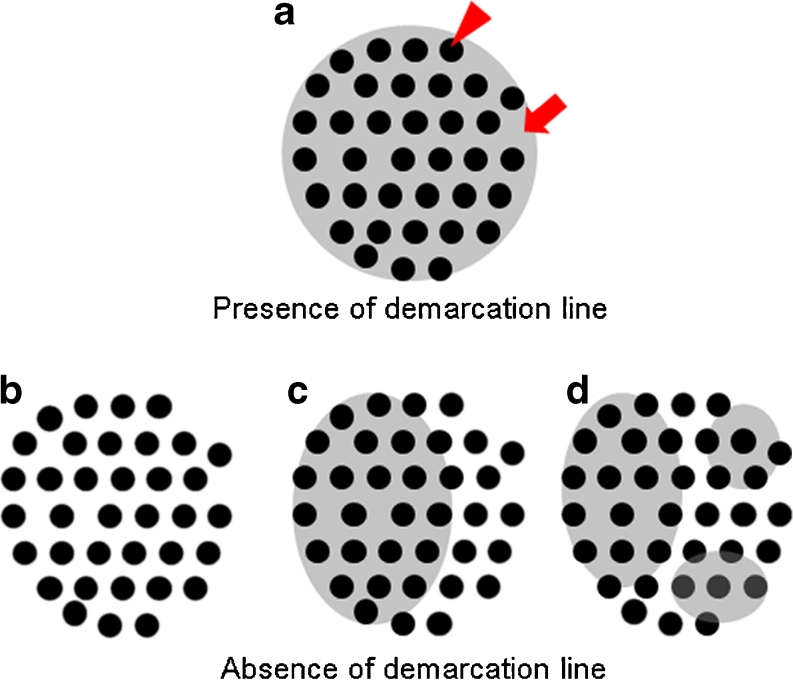
**a** Presence of demarcation line: uniform distribution of brownish epithelium (*arrow*) over the entire lesion in the presence of brownish dots (*arrowhead*). **b** Absence of demarcation line: no brownish epithelium. **c** Absence of demarcation line: partial distribution of brownish epithelium in the presence of brownish dots. **d** Absence of demarcation line: nonuniform distribution of brownish epithelium over the entire lesion

We did not perform iodine staining or biopsy in the diagnosis of 53 MSENs before EMR in this study. ME-NBI of the 53 MSENs revealed brownish dots, brownish epithelium, and demarcation line. Brownish dots indicated dilated, extended, and tortuous IPCL with caliber variation and dilation. Epithelium was recognized as the brownish area in each IPCL. Demarcation line was observed as uniformly distributed brownish epithelium over the area of brownish dots. The absence of demarcation line was indicated by no or only partially visible brownish epithelium, or nonuniform distribution of brownish epithelium in the area of brownish dots, as shown in Fig. 1.

The endoscopists who examined ME-NBI were not blind to clinical details of all patients. Intraobserver agreement was evaluated at 1-month intervals and interobserver agreement was evaluated between two experienced endoscopists (Y.M. and Y.S.) who both had used the NBI system for more than 5 years. They were blind to the patients’ final clinical information and histological results of the lesions. Intra- and interobserver agreement were assessed by kappa statistics.

### Pathological Examination

The resected samples were diagnosed by hematoxylin–eosin staining. Only one pathologist in our hospital diagnosed all the lesions using an electron microscope and classified the lesions according to the World Health Organization classification [19]. Histopathological type [low-grade intraepithelial neoplasia (LGIN), high-grade intraepithelial neoplasia (HGIN), and squamous cell carcinoma (SCC)], depth of invasion, lateral and vertical resection margin, and level of lymphovascular involvement were microscopically evaluated.

## Results

The characteristics (age, sex, smoking, and drinking habits) of patients who underwent EMR in this study are shown in Table 1. Mean age of the 40 patients was 69.5 years (range 51–80) and the male to female ratio was 34:6. Smoking habits were measured by the Brinkman index (number of cigarettes smoked per day × number of years of smoking), and drinking habits were shown by average daily amount of alcohol intake (gram). The average Brinkman index score was 880 (range 0–3,360). The average daily amount of alcohol intake was 61 g (range 0–292). All 53 lesions in this study were excised by EMR. No complications such as perforation and bleeding were observed. All lesions had negative surgical margins. No lymphatic or vascular invasion was evident. No recurrence has been observed to date.

**Table 1:** Baseline characteristics of patients

	*n* = 40
Age (years)	
Median (range)	69.5 (51–80)
Gender	
Male/female	34/6
Brinkman index	
Median (range)	880 (0–3,360)
Daily alcohol intake (g)	
Median (range)	61 (0–292)

Smoking habit was shown by Brinkman index (number of cigarettes smoked per day × smoking years), and drinking habit shown by average daily amount of alcohol intake (gram)

Histopathological examination identified six lesions (11.3 %) as SCC in the LPM. Other classifications were as follows: 47 lesions (88.7 %) were intraepithelial neoplasias, 35 (66.0 %) were LGIN, and 12 (22.6 %) were HGIN. No inflammation or other neoplastic lesions were observed. Characteristics (tumor size, localization, morphology) of the resected lesions are shown in Table 2 for the three groups: LGIN, HGIN, and SCC. Average tumor sizes in the LGIN, HGIN, and SCC groups were 3.1, 5.4, and 7.2 mm, respectively. Localization of tumors was as follows: upper: 14, middle: 20, lower: 1 for LGIN; upper: 3, middle: 8, lower: 1 for HGIN; and upper: 2, middle: 2, lower: 2 for SCC. All 53 lesions appeared as brownish areas on ME-NBI. Morphologically, four lesions (HGIN—two, SCC—two) were slightly depressed and the other 49 lesions were all flat type.

**Table 2:** Characteristics of the endoscopically resected small esophageal lesions

	LGIN (*n* = 35)	HGIN (*n* = 12)	SCC (*n* = 6)
Tumor size (mm)			
Mean (SD)	3.1 (1.8)	5.4 (2.3)*	7.2 (2.5)**
Tumor location			
Upper	14	3	2
Middle	20	8	2
Lower	1	1	2
Tumor morphology			
0-IIa	0	0	0
0-IIb	35	10	4
0-IIc	0	2	2

*LGIN* low-grade intraepithelial neoplasia, *HGIN* high-grade intraepithelial neoplasia, *SCC* squamous cell carcinoma, *0-IIa* slightly elevated type, *0-IIb* flat type, *0-IIc* slightly depressed type

*P < 0.05; **P < 0.005: *P* value was calculated as comparisons between LGIN, HGIN, LGIN, and SCC

*P* values of <0.05 on two-tailed Welch’s *t* test were considered statistically significant. HGIN was significantly greater than LGIN (*P* = 0.0039). SCC was also significantly greater than LGIN (*P* = 0.015).

Results of ME-NBI for MSEN in this study are shown in Table 3. *P* values of <0.05 in two-tailed Fisher’s exact test were considered statistically significant. Significantly more brownish dots and brownish epithelium were observed in HGIN compared with LGIN (*P* = 0.037 and *P* = 0.0014, respectively). No significant difference in demarcation line was observed between the three groups.

**Table 3:** NBI findings in small esophageal lesions

	LGIN (*n* = 35)	HGIN (*n* = 12)	SCC (*n* = 6)	*P* value
Brownish dots				0.037
Positive	20	11	5	
Negative	15	1	1	
Brownish epithelium				0.0014
Positive	17	12	5	
Negative	18	0	1	
Demarcation line				
Positive	13	7	5	
Negative	22	5	1	

*P* value of <0.05 was considered statistically significant (two-tailed Fisher’s exact test)

*LGIN* low-grade intraepithelial neoplasia, *HGIN* high-grade intraepithelial neoplasia, *SCC* squamous cell carcinoma

As shown in Fig. 1, a demarcation line was observed as a uniform distribution of brownish epithelium over the entire area of brownish dots. The absence of a demarcation line was indicated when brownish epithelium was not visible or only partially visible, or distribution of brownish epithelium in the area of brownish dots was not uniform. Lesions without a demarcation line were found in all three groups as follows: LGIN (*n* = 4), HGIN (*n* = 5), and SCC (*n* = 1). When MSEN was diagnosed as HGIN or SCC, sensitivity, specificity, positive predictive value, and negative predictive value were 88.9, 42.9, 44.4, and 88.2 %, respectively, for brownish dots; 94.4, 51.4, 50.0, and 94.7 %, respectively, for brownish epithelium; and 66.7, 62.9, 48.0, and 78.6 %, respectively, for demarcation line (Table 4).

**Table 4:** Sensitivity, specificity, and predictive values of NBI findings in small esophageal lesions, when MSEN was diagnosed as high-grade intraepithelial neoplasia or squamous cell carcinoma

	Sensitivity (%)	Specificity (%)	PPV (%)	NPV (%)
Brownish dots	88.9	42.9	44.4	88.2
Brownish epithelium	94.4	51.4	50	94.7
Demarcation line	66.7	62.9	48	78.6

*PPV* positive predictive value, *NPV* negative predictive value, *MSEN* minimal superficial esophageal neoplasia

Intraobserver agreement (*κ* value) of brownish dots, brownish epithelium, and demarcation line was 0.78, 0.72, and 0.61, respectively, which indicated substantial agreement. Interobserver agreement (*κ* value) of brownish dots, brownish epithelium, and demarcation line was 0.58, 0.52, and 0.41, respectively, which indicated moderate agreement.

**Fig. 2:**
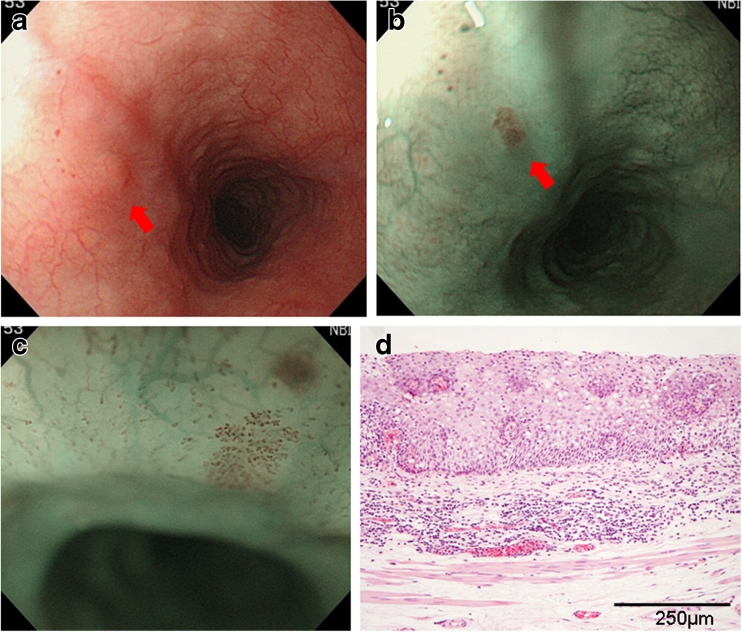
**a** A case of LGIN. Note the flat reddened area 2 mm in diameter on white light in the upper esophagus (*arrow*). **b** Brownish area on NBI (*arrow*). **c** Extended IPCL visible by ME-NBI. **d** Resected specimen of LGIN. Neoplastic cells with atypical nuclei and prominent nucleoli are present in <50 % of the thickness of the epithelium (hematoxylin and eosin stain)

**Fig. 3:**
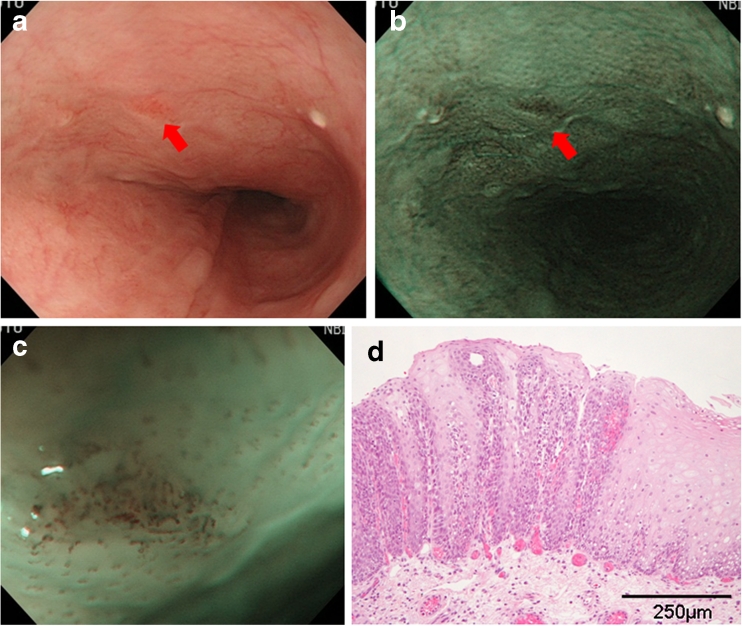
**a** A case of HGIN. Note the flat reddened area 2 mm in diameter on white light in the middle esophagus (*arrow*). **b** Brownish area on NBI (*arrow*). **c** Dilated, extended, and tortuous IPCL with brownish epithelium visible by ME-NBI. **d** Resected specimen of HGIN. Neoplastic cells with atypical nuclei and prominent nucleoli in >50 % of the thickness of the epithelium (hematoxylin and eosin stain)

**Fig. 4:**
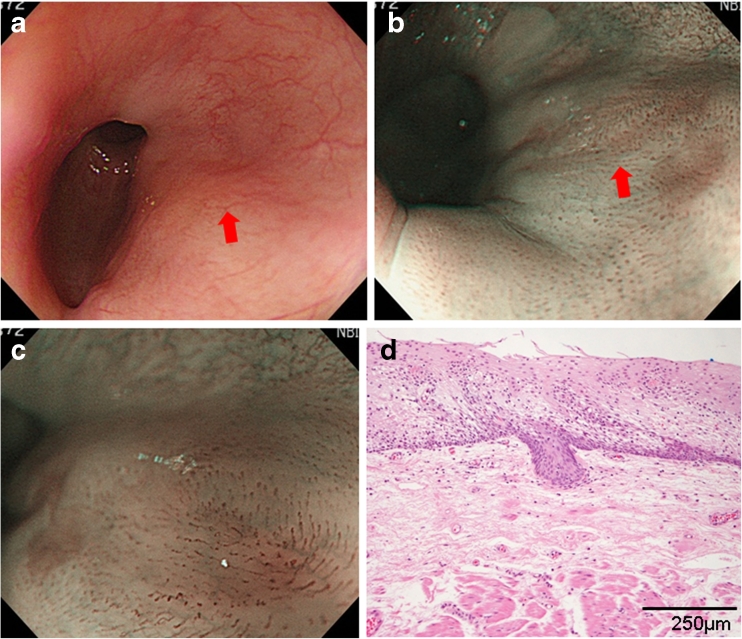
**a** A case of SCC. Note the flat reddened area 9 mm in diameter with white light in the lower esophagus (*arrow*). **b** Brownish epithelium with irregular borderline (*arrow*). **c** Dilated and extended IPCL with well-demarcated brownish area visible by ME-NBI. **d** Resected specimen of SCC. Neoplastic cells are present throughout the epithelium and invade the LPM (hematoxylin and eosin stain)

Endoscopic and histopathological findings in patients with typical LGIN, HGIN, and SCC diagnosed by NBI are shown in Figs. 2, 3, and 4, respectively.

Figure 2 shows the case of a 66-year-old woman with a flat reddened area 2 mm in diameter at the upper esophagus visible by conventional endoscopy with white light (Fig. 2a arrow). The lesion appeared as a brownish area on NBI (Fig. 2b arrow) and was diagnosed as esophageal epithelial neoplasia with enlarged and tortuous IPCL by ME-NBI (Fig. 2c). After EMR, histopathological examination of the resected lesion led to a diagnosis of LGIN (Fig. 2d) because neoplastic cells with atypical nuclei and prominent nucleoli were present in <50 % of the thickness of the epithelium.

Figure 3 shows the case of a 65-year-old woman with a flat reddened area 2 mm in diameter in the middle esophagus visible by conventional endoscopy with white light (Fig. 3a arrow). The lesion appeared as a brownish area on NBI (Fig. 3b arrow) and was diagnosed as esophageal epithelial neoplasia with dilated, enlarged, and tortuous IPCL and brownish epithelium by ME-NBI (Fig. 3c). After EMR, histopathological examination of the resected lesion led to a diagnosis of HGIN (Fig. 3d) because neoplastic cells with atypical nuclei and prominent nucleoli were present in >50 % of the thickness of the epithelium.

Figure 4 shows the case of a 79-year-old man with a flat reddened area 9 mm in diameter with poor diaphanous vessels at the lower esophagus visible by conventional endoscopy with white light (Fig. 4a arrow). The lesion appeared as a well-demarcated brownish area on NBI (Fig. 4b arrow) and was diagnosed as esophageal epithelial neoplasia with dilated, enlarged, and tortuous IPCL with caliber variation on ME-NBI (Fig. 4c). After EMR, pathological examination of the resected lesion led to a diagnosis of SCC because neoplastic cells were present throughout the epithelium and invaded the LPM with negative lateral margins and without lymphatic and vascular invasion (Fig. 4d).

## Discussion

NBI is an optical technique that is implemented by dedicated CLV-260SL or CV-260SL source units (Olympus Optical Co., Ltd., Tokyo, Japan). There are many different scopes available. Using an optical filter, NBI utilizes the absorption characteristics of hemoglobin, generating light in two narrow bands of 540 and 415 nm. Hemoglobin strongly absorbs these wavelengths. Light of these wavelengths is suitable for visualizing the microvessels and microstructure of gastrointestinal mucosa. The light is propagated at different depths in the mucosal tissues, which enables detailed description of blood vessel morphology. The 415-nm narrow band of light depicts surface vessels of the mucosa as brown, and the 540-nm narrow band depicts subepithelial vessels as cyan. The color difference enables definitive representation of microvessels on images.

Conventionally, iodine staining is useful in the diagnosis of esophageal cancer [11], but it presents certain problems. Iodine irritates the esophagus, and considerable effort is necessary to insert the spray tube. In contrast, NBI is an optical image enhancement technology that features a simple one-touch system and can be performed without irritation to the esophagus or the hassle of dye spray. One report indicated no difference between NBI and iodine staining in the diagnosis of HGIN [20]. In the series reported here, NBI was clinically easy to perform and more useful than iodine staining. Therefore, in screening endoscopy in our hospital, NBI has been used more frequently than iodine staining, and it is now a standard procedure in almost all cases since its introduction.

Morphology if MSENs <10 mm in diameter included in this study was mostly flat. These small MSENs were generally recognized by their slightly red color on conventional endoscopy with white light. However, they were more easily recognized by their brownish color on NBI. Esophageal epithelial tumors appear brownish on NBI and are identified by crowding of dilated IPCL and brown epithelium between IPCLs [21]. Thus, NBI enabled detection of very small esophageal lesions. Shimizu et al. reported that 0-IIb lesions <1 cm in the largest diameter of the esophagus might be regarded as intraepithelial neoplasia [22]. This was true in our study, which was limited to lesions <10 mm in diameter. However, although we examined esophageal epithelial tumors <10 mm, SCC was also found (6/53, 11.3 %). Because small esophageal lesions are sometimes also identified as SCC, we examined the diagnostic performance of ME-NBI, instead of the ability of this combined technique to differentiate tumor size.

It is important to detect ESCC early, when it is small, to facilitate early treatment. However, there has been some debate as to whether it is really important to detect LGIN or HGIN because of treatment difficulties. One report stated that 30 % of lesions diagnosed as HGIN by biopsy had been diagnosed pathologically as SCC after EMR [22]. Because we have had similar experiences in our hospital, we consider HGIN to be treatable.

In intraepithelial neoplasia of the esophagus, it is important to discriminate between LGIN and HGIN. Muto et al. argued that the brownish epithelium visible on NBI could differentiate the boundaries to some extent in distinguishing between LGIN and HGIN [21].

In the study of Ishihara et al., sensitivity and specificity in diagnosing HGIN and SCC from brownish dots and brownish epithelium visible on NBI were 85 and 79 %, respectively [23]. According to their study, brownish dots and brownish epithelium were significant NBI findings in esophageal squamous mucosal high-grade neoplasia and demarcation line was not a statistically significant finding. But we also re-evaluated demarcation line, because demarcation line was significantly associated with the diagnosis of mucosal high-grade neoplasia in univariate analysis by Ishihara et al., and we considered that it could be a simple index in diagnosing HGIN and SCC by redefining it as uniformly distributed brownish epithelium over the area of brownish dots. The same trend of significantly more brownish dots and brownish epithelium in HGIN and SCC was observed in this study of lesions <10 mm in diameter.

Considering the results of the combined technique in terms of sensitivity, specificity, and predictive value, MSEN with neither brownish dots nor brownish epithelium seemed likely to be LGIN. In addition, ME-NBI of MSEN seemed to be useful to some extent in distinguishing between lesions that need to be treated, such as those classified as HGIN or SCC, and LGIN lesions, which may not require immediate treatment. In other words, ME-NBI was useful in determining indications for treatment of MSEN.

However, LGIN can develop to HGIN and SCC if left untreated. Because even small lesions turn out to be HGIN after resection, and lesions diagnosed as HGIN by NBI turn out to be LGIN, we conclude that resection of LGIN in the cases examined here was warranted. In this study, written consent was obtained from all patients before EMR.

Detection of minimal LGIN and HGIN is very useful for the following reasons: These types of neoplasias carry significant risk of progression to SCC [24]. In addition, lugol voiding lesions (LVLs), including intraepithelial neoplasias of varying degrees, are an important risk factor for a second primary SCC in patients with esophageal cancer or head and neck cancer [25]. The larger the size of the unstained area by iodine staining, the higher the frequency of HGIN and carcinoma in situ than LGIN [26]. The presence of multiple LVLs in patients with head and neck cancer is an independent risk factor for multifocal ESCC [27].

As with multiple LVLs detected by iodine staining, detecting multiple LGINs and HGINs of smaller size by NBI and identifying risk factors in the development of SCC is clinically useful. However, different levels of experience with NBI may lead to significant differences in lesion detection [28]. Thus, sufficient training is needed.

In conclusion, NBI combined with ME contributed to accurate detection of MSEN. ME-NBI enabled differential diagnosis of MSEN. Limitations of this study included its single-center, retrospective nature, and small number of cases. There was also variation in endoscope selection and skill level of the operators.
